# Novel use of dupilumab in pemphigus vulgaris and pemphigus foliaceus

**DOI:** 10.1016/j.jdcr.2023.09.018

**Published:** 2023-09-30

**Authors:** Christina Jiang, Susuana Adjei, Sueheidi Santiago, Jun Lu, Miguel Duran, Stephen Tyring

**Affiliations:** a Department of Dermatology, University of Connecticut Health Center, Farmington, Connecticut; b Department of Dermatology, University of Texas Health Science Center, Houston, Texas

**Keywords:** dupilumab, immunobiologic, pemphigus vulgaris, pemphigus foliaceus

## Introduction

Pemphigus vulgaris (PV) and pemphigus foliaceus (PF) belong to a family of autoimmune dermatoses of mucous and/or cutaneous membranes in which acantholysis leads to bullae and erosion formation.^1^^,^^2^ The pathogenesis of PV and PF involve the antibodies against desmogleins, transmembrane glycoproteins associated with desmosomes that mediate cell–cell adhesion within the epidermis.^1^^,^^2^ Antibodies against desmogleins 1/3 are involved in PV, and only antibodies against desmoglein 1 are involved in PF, which contribute to differences in clinical presentation.^1^^,^^2^

PV and PF are rare with high morbidity in affected patients. The prognosis has improved with current treatment options of systemic glucocorticoids alone and/or steroid-sparing treatments, such as rituximab, mycophenolate mofetil, or azathioprine.^1^ Prolonged systemic glucocorticoid therapy and systemic immunosuppressants can lead to side effects, including metabolic derangements, osteoporosis, immunosuppression, etc^1^ so the benefits need to be weighed with risks.

Dupilumab is a monoclonal antibody blocking interleukin 4/13 receptor-α, currently US Food and Drug Administration–approved for asthma, atopic dermatitis, eosinophilic esophagitis, prurigo nodularis, and rhinosinusitis with nasal polyposis.^3^ Multiple case reports have suggested that dupilumab might be an effective treatment with minimal side effects for moderate and severe bullous pemphigoid as steroid-sparing agents, either alone or together with other immunomodulators.^4^^,^^5^ There is an ongoing phase 2 double-blinded randomized clinical trial on dupilumab for bullous pemphigoid, but whether dupilumab may improve pemphigus is unclear. Only 1 case report has been published on dupilumab significantly improving PV after failed topical and systemic corticosteroids.^6^ We describe 3 patients with PV or PF who responded to dupilumab after failing multiple therapies over a minimum of 4 years since the initial diagnosis.

## Case 1

A 55-year-old Asian American woman with a 4-year history of poorly controlled PF presented to our clinic with multiple scaly and ulcerated plaques and flaccid bullae located on the neck, chest, back, and scalp. There was no mucosal involvement. Diagnosis of PF was confirmed with histopathology, and indirect and direct immunofluorescence. Indirect immunofluorescent (IIF) was positive for desmoglein 1. Direct immunofluorescence showed intercellular IgG and negative for immunoglobulin M, IGA, and C3. She had failed multiple courses of systemic corticosteroids plus potent topical corticosteroid. Two weeks prior to her presentation, she had just finished another course of oral prednisone 20 mg tapering over the course of 20 days. She was also using clotrimazole/betamethasone cream twice a day for 1 month without improvement. She was then initiated on dupilumab with a loading dose of 600 mg followed by 300 mg subcutaneous injection every 2 weeks in addition to topical corticosteroid. She had marked improvement in her cutaneous manifestation with complete resolution of painful erosions and bulla after 60 weeks of dupilumab. She continued to be in clinical remission beyond 60 weeks and is currently on dupilumab 300 mg every 8 weeks as maintenance.

## Case 2

**Fig 1:**
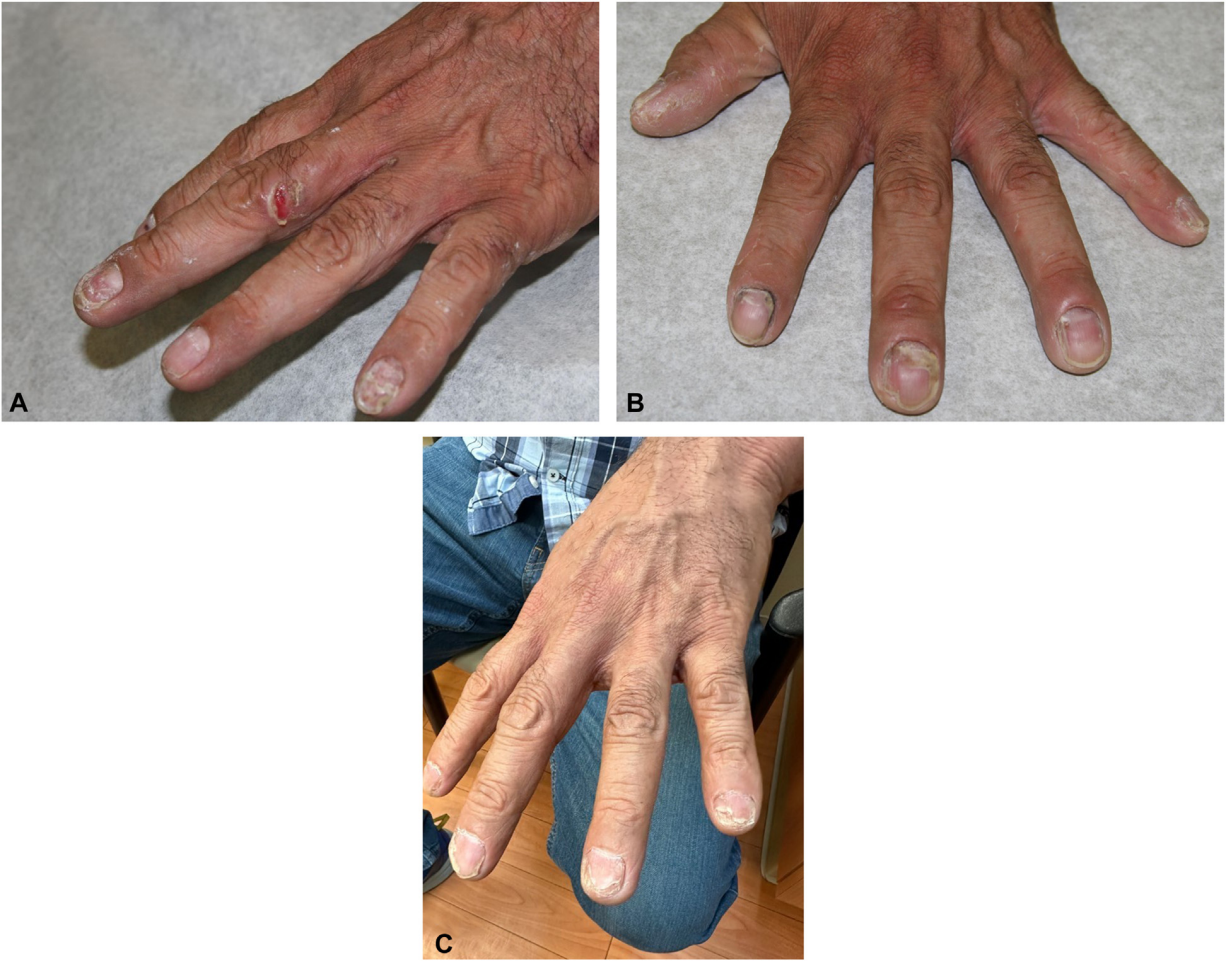
Case 2 - pemphigus vulgaris of left hand. **A,** Baseline of left hand: eroded plaque on the left lateral third digit**. B,** Left hand after 20 weeks of dupilumab: clinical remission**. C,** Left hand after 63 weeks of dupilumab: maintenance of clinical remission

**Fig 2:**
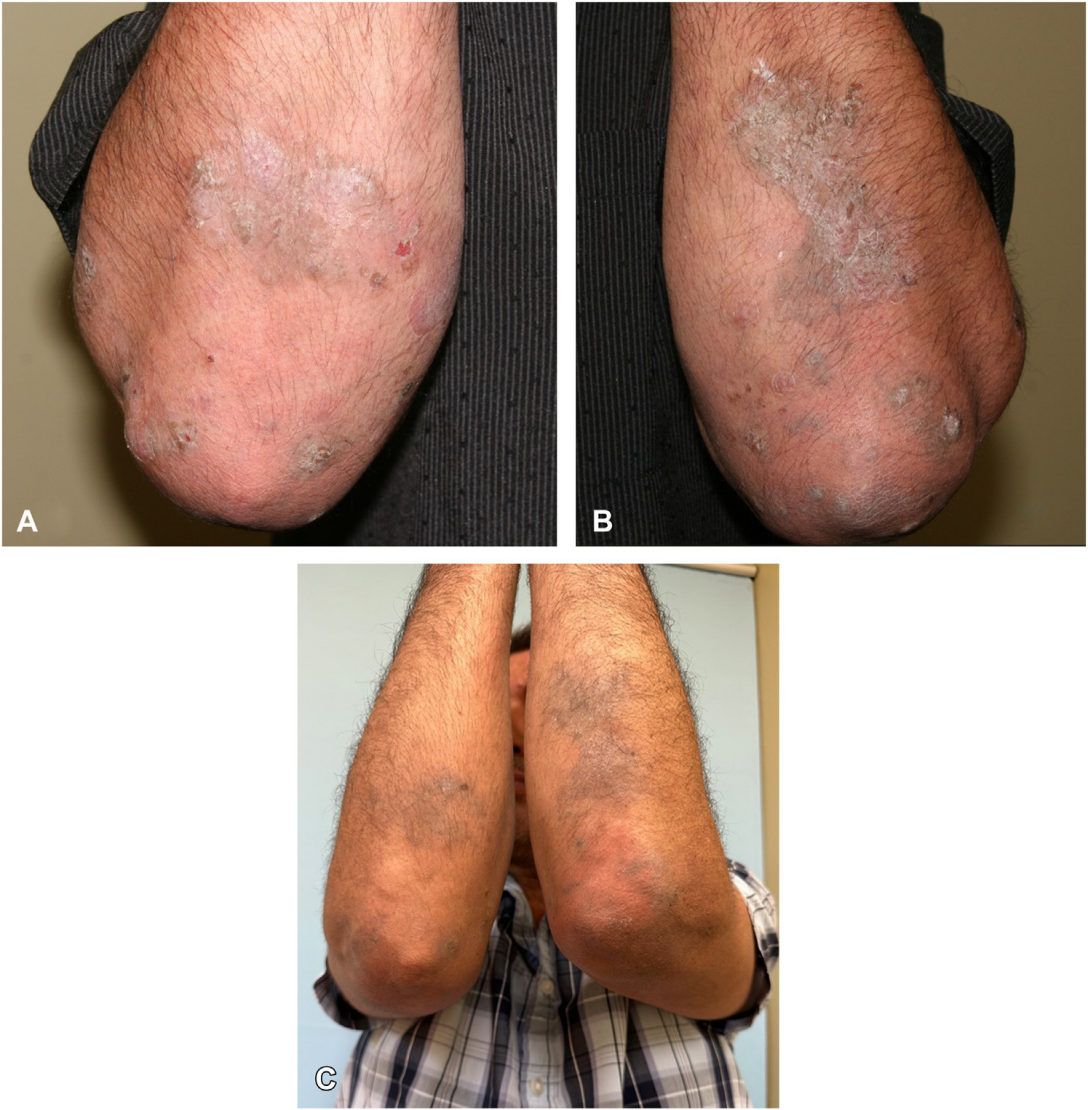
Case 2 - pemphigus vulgaris of forearms. **A,** Baseline of dorsal aspect of the right forearm: scaly brown plaques with eroded bullae**. B,** Baseline of dorsal aspect of the left forearm: scaly brown plaques with eroded bullae**. C,** Bilateral dorsal aspect of the forearms after 40 weeks of dupilumab: scaly brown plaques that are decreased in size from baseline images, no bullae or ulcerations.

**Fig 3:**
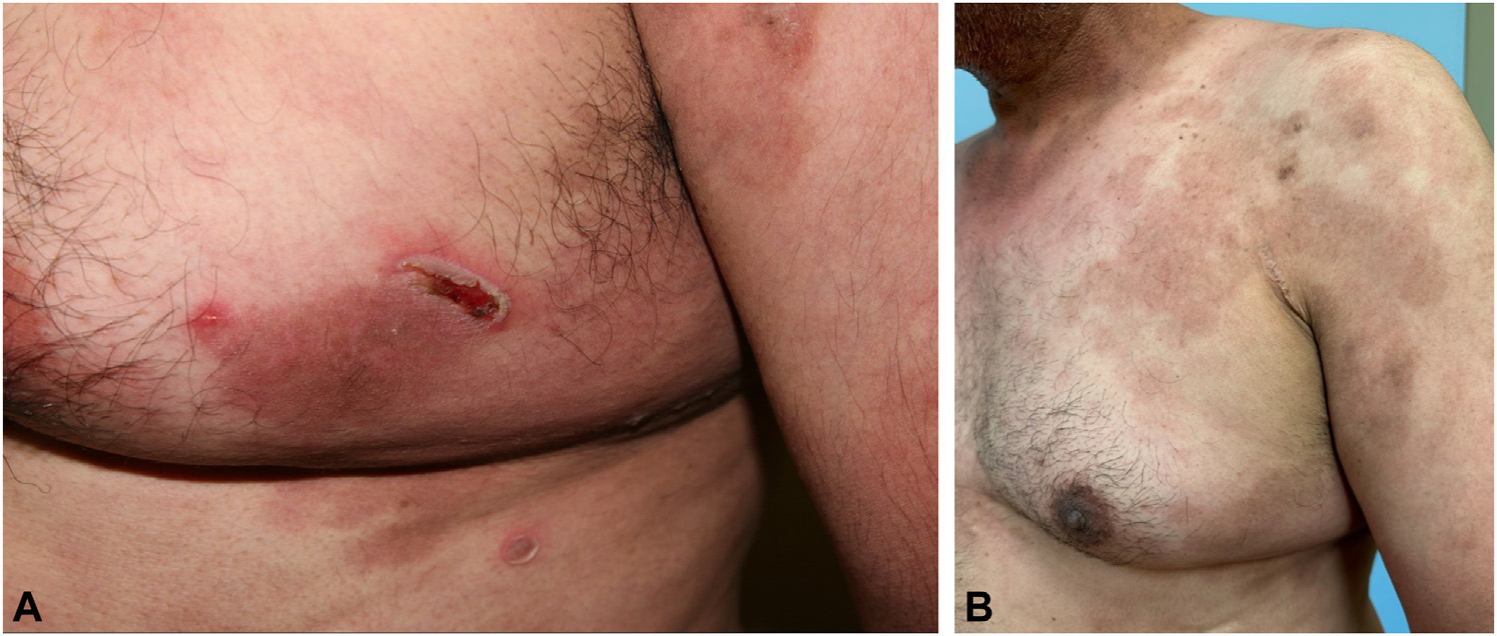
Case 2 - pemphigus vulgaris of left breast. **A,** Baseline of left breast: brown plaque with eroded bullae**. B,** Left breast after 63 weeks of dupilumab: postinflammatory hyperpigmentation without bullae.

A 47-year-old Hispanic otherwise healthy man diagnosed with PV affecting oral mucosa, face, hands, arms, abdomen, and left breast. Patient had eroded plaques affecting his left hand (Fig 1, *A*), scaly brown plaques with eroded bullae on his dorsal aspect of the forearms (Fig 2, *A* and *B*), and brown plaque with eroded bullae on his left breast (Fig 3, *A*). Diagnosis was confirmed with skin biopsy for hematoxylin-eosin stain and direct immunofluorescence as well as serology for IIF. He did not respond to rituximab, prednisone tapers, mycophenolate mofetil, and topical tacrolimus, and painful erosions continued to develop. On presentation, patient has finished his rituximab infusion 1 month ago and was on mycophenolate mofetil at 500 mg 3 times a day. We then switched mycophenolate mofetil to dupilumab 300 mg subcutaneously every 2 weeks. Although he developed 2 small bullae 3 days after dupilumab initiation, they resolved quickly in 1 week. He had marked improvement and complete resolution of erosions on his hands (Fig 1, *B*), after 20 weeks of treatment. He had complete resolution of bullae and erosion on bilateral forearms after 40 weeks of treatment with postinflammatory hyperpigmentation. He continued to maintain clinical remission after 63 weeks of dupilumab treatment (Figs 1, *C*; 2, *C*; and 3, *B*). He currently remains on dupilumab 300 mg every 6 weeks.

## Case 3

An 80-year-old Asian American man with history of hyperlipidemia was diagnosed with PV affecting the scalp and oral mucosa. Patient had painful ulcers in the oral cavity and ulcerated plaques with occasional flaccid bullae on the scalp. Diagnosis was confirmed with biopsy for hematoxylin-eosin stain and direct immunofluorescence and serology for IIF. IIF was positive for both desmogleins 1 and 3. He failed treatment with oral prednisone, azathioprine, and topical fluocinonide. Due to lack of response to conventional therapies, he was started on dupilumab at a dose of 300 mg subcutaneously every 2 weeks in addition to prednisone taper, azathioprine, topical tacrolimus, clobetasol, and benzocaine. Unfortunately, he started to develop new painful mouth ulcers and blisters on the right side of the scalp, shoulders, and back 4 weeks after initiation of dupilumab, which resulted in its discontinuation.

## Discussion

This retrospective case series from 2 academic institutions evaluates the outcomes of 3 patients with PV or PF treated with dupilumab; 2 men and 1 woman with an age range of 47 to 80 years. All 3 patients failed systemic and topical corticosteroid treatments and/or other nonsteroidal treatments prior to dupilumab therapy. All patients were started on dupilumab at least 4 years after their initial diagnosis. All patients received concomitant immunomodulating medications. Two patients had mild-to-moderate disease flare after dupilumab initiation, which is likely associated with discontinuation of prednisone. No adverse events were reported during therapy. Two patients had complete or marked response after treatment, whereas case 3 had none to minimal response and discontinued dupilumab. It is unclear whether this patient might benefit from a longer treatment length. Nonetheless, patients 1 and 2 who responded to dupilumab maintained their disease control for at least 60 weeks of dupilumab treatment and were able to sustain treatment response at decreased frequency of dupilumab, 8 and 6 weeks, respectively.

Similar to that in bullous pemphigoid, the autoimmune reaction in PV and PF is believed to be driven by T helper 2 cells. Interleukin 4 can exacerbate T helper 2 overexpression and isotype switching to IgG1 and IgG4 responsible for desmoglein loss.^4^ This provides a potential mechanism for the use of dupilumab in PV and PF. Limitations to this study include its retrospective nature, low number of patients, and lack of a control group. More clinical studies and case reports are needed to further explore the potential of dupilumab as a safe steroid-sparing therapy for pemphigus.

## Conflicts of interest

Drs Tyring and Lu have worked on clinical trials with Regeneron, but this study was self-funded. Drs Jiang, Adjei, Santiago, and Duran have no conflicts of interest to declare.
